# Correcting the Cystic Fibrosis Disease Mutant, A455E CFTR

**DOI:** 10.1371/journal.pone.0085183

**Published:** 2014-01-08

**Authors:** Liudmila Cebotaru, Daniele Rapino, Valeriu Cebotaru, William B. Guggino

**Affiliations:** 1 Department of Ophthalmology, School of Medicine, The Johns Hopkins University, Baltimore, Maryland, United States of America; 2 Department of Physiology, School of Medicine, The Johns Hopkins University, Baltimore, Maryland, United States of America; 3 Department of Medicine, School of Medicine, The Johns Hopkins University, Baltimore, Maryland, United States of America; National Center for Scientific Research Demokritos, Greece

## Abstract

Cystic fibrosis is caused by more than 1000 mutations, the most common being the ΔF508 mutation. These mutations have been divided into five classes [1], with ΔF508 CFTR in class II. Here we have studied the class V mutation A455E. We report that the mature and immature bands of A455E are rapidly degraded primarily by proteasomes; the short protein half-life of this mutant therefore resembles that of ΔF508 CFTR. A455E could be rescued by treatment of the cells with proteasome inhibitors. Furthermore, co-transfection of A455E with the truncation mutant Δ264 CFTR also rescued the mature C band, indicating that A455E can be rescued by transcomplementation. We found that Δ264 CFTR bound to A455E, forming a bimolecular complex. Treatment with the compound correctors C3 and C4 also rescued A455E. These results are significant because they show that although ΔF508 belongs to a different class than A455E, it can be rescued by the same strategies, offering therapeutic promise to patients with Class V mutations.

## Introduction

Cystic fibrosis (CF) is caused by mutations in the cystic fibrosis transmembrane conductance regulator (CFTR) [2]. Symptoms of CF include higher-than-normal sweat chloride, thick airway mucus, persistent lung infections, pancreatic enzyme insufficiency, intestinal blockage, and infertility in males [3]. These classic symptoms of CF can range in severity from mild to severe. Extensive effort has been made to understand the genotype of CF patients, with over 1000 gene mutations identified thus far [4]. These mutations in the CF gene have been divided into five different classes: Class I mutations result in defective protein production. Class II mutations result in a protein whose processing is blocked in the ER. The most common CFTR mutation, ΔF508 CFTR [2], is a class II mutation. Like other class II mutations, ΔF508 CFTR is retained in the ER, incompletely glycosylated, and rapidly degraded in proteasomes [5]. Class III mutations produce a protein that has defective regulation; the most common is the G551D mutation, which reaches the cell surface but does not conduct chloride [6], [7]. Class IV mutations cause defects in channel conductance. Finally, class V mutations affect protein synthesis or splicing, causing less protein to be made. One of these class V mutations is A455E.

The A455E mutation is located in NBD1. It was originally found in French Canadian patients and is associated with a mild phenotype, with borderline high sweat, moderate lung disease, and sufficient pancreatic function [8], [9]. Unlike other mild missense mutations such as R117H that have altered channel conductance [10] and are considered class IV mutations, the single-channel characteristics of A445E resemble those of wild-type CFTR [11], [12] . Thus, because the mild disease resulting from A455E is thought to arise from reduced protein expression, it is considered a class V mutation. Thus, an effective pharmacological approach to treating this mutation should involve increasing the protein levels of A455E.

Our group has been interested in transcomplementation [13], [14] using Δ264 CFTR, which is a truncated version of CFTR missing the first four transmembrane domains. When monkey lungs are infected with an adeno-associated viral vector rAAV-Δ264 CFTR, the Δ264 CFTR produced can increase the levels of endogenous wild-type CFTR protein [15]. We have also shown in cotransfection studies that Δ264 CFTR increases wild-type CFTR protein levels and increases the degree of maturation of the immature band B to the mature C band of ΔF508 CFTR. The purpose of the current study was to determine whether analogous transcomplementation can be used to enhance the protein processing of A455E.

## Experimental Procedures

### Cell culture

African green monkey kidney cells (Cos7) were maintained in Dulbecco’s modified Eagle’s medium-high glucose 1x (DMEM), penicillin (100 U/ml), streptomycin (100 µg/ml), and 10% fetal bovine serum as described previously [14]).

### Plasmids and constructs

The construct pEGFP A455E was a gift from Dr. Gary Cutting at Johns Hopkins U. The plasmids were transfected into Cos 7 cells using Lipofectamine 2000 (Invitrogen) as we have previously described. After 48 h of transfection, the cells were harvested and used for immunoprecipitation and immunoblotting.

### Immunoblotting and immunoprecipitation

Cells were harvested and processed as described previously [16] using the (C-terminus) antibody (1:1500; R&D Systems, Inc.). Glyceraldehyde 3-phosphate dehydrogenase (GAPDH), used as a loading control, was detected with monoclonal anti-GAPDH antibody (1∶10,000; US Biological).

For immunoprecipitation, cells were harvested and processed as described previously. For pull-down experiments, 10 µl of anti-GFP antibody (Roche) were added to the lysate and allowed to incubate for 30 min. with 50 µl of A/G-agarose beads (Santa Cruz Biotechnology, Inc.). CFTR was detected as described above.

### Statistics

Western blots were evaluated by one-way ANOVA followed by LSD post hoc tests. Statistical significance was set at P<0.05, and data are presented as mean±2SEM. All experiments were normalized for the control. SPSS (version 17.0 SPSS, Inc, Chicago, III) was used for data analysis

## Results

### Expression of A455E

**Figure 1:**
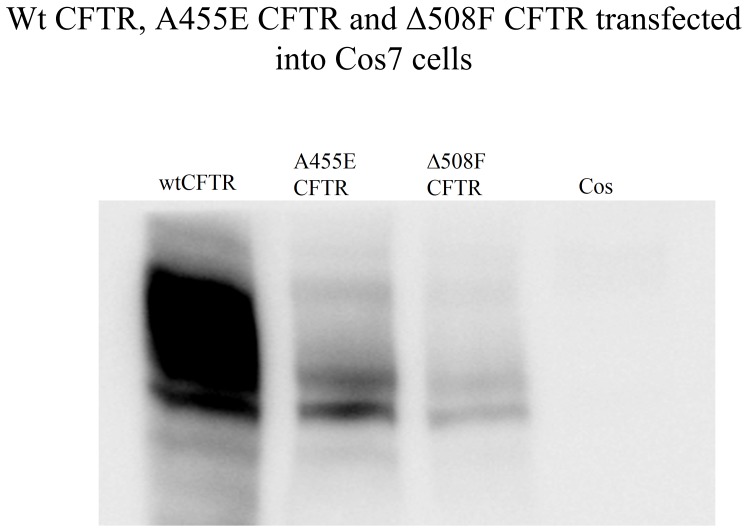
A455E has reduced expression of mature CFTR. Cos7 cells were transfected with 2 µg of wild-type, A455E, or ΔF508 CFTR constructs. After 48 h, the cells were lysed, and the total lysate was analyzed by western blotting with anti-human CFTR antibodies. Note that there is much less mature C band in the A455E sample than in the wild-type sample. In this and subsequent blots, some mature C band was detected with A455E (n = 8).

**Figure 2:**
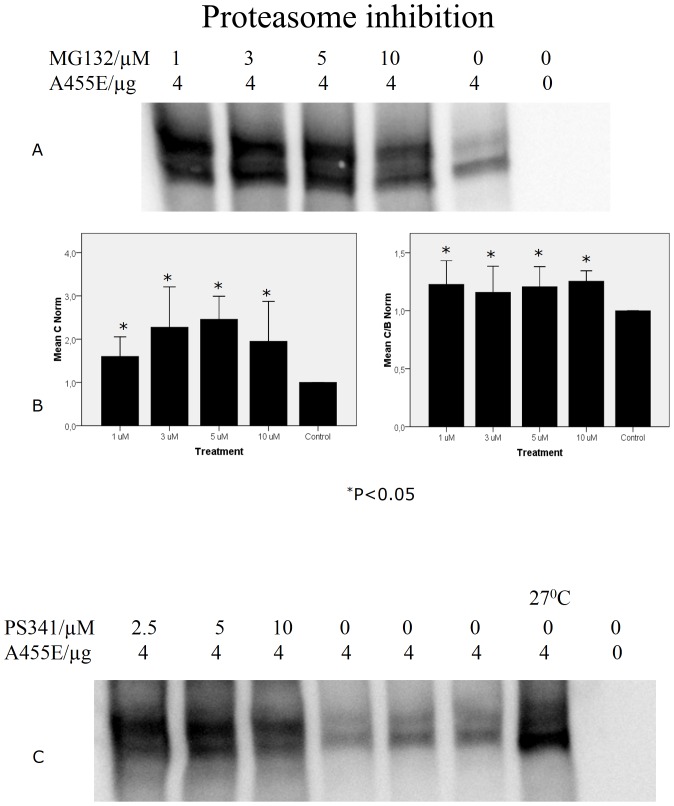
Proteasome inhibition. Cells transfected with A455E cDNA were treated either with MG132, a more general inhibitor (n = 6) (**A, B**), or the more specific inhibitor of proteasomes, PS341 (n = 2) (**C**). Note that in both cases, proteasome inhibition caused an increase in both the immature B and mature C bands of A455E.

**Figure 3:**
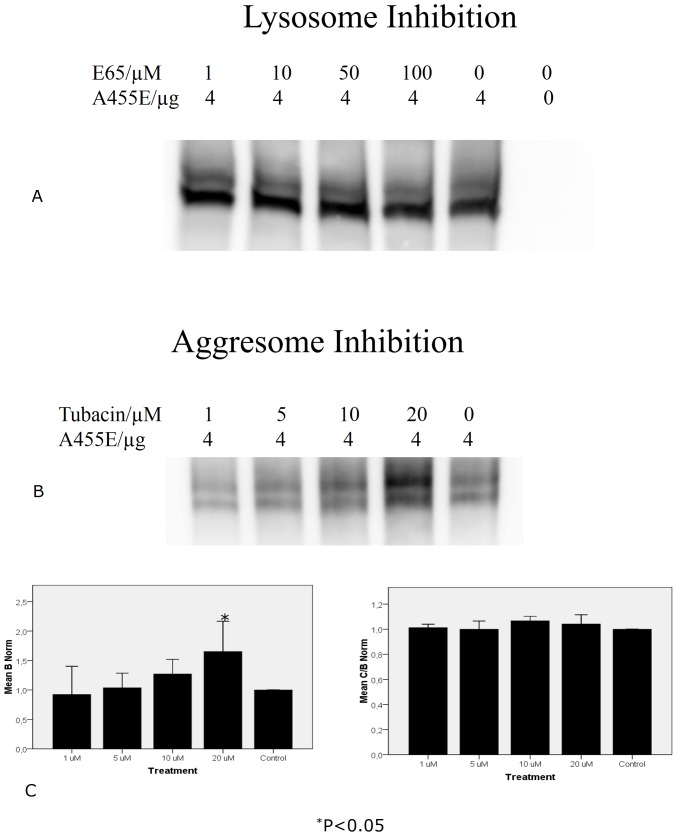
Lysosome/aggresome inhibition. Cos7 cells were transfected with A455E CFTR cDNA and treated for 16 h with the lysosome inhibitor E64 (n = 4) (**A**) There was very little change in band density in any of the treated groups or in the presence of the inhibitors when compared to the control or the HDAC6 inhibitor tubacin to inhibit aggresomes (n = 3) (**B, C**). Note that there is a significant increase with the highest concentration used.

When we compared the expression of the A455E mutant to that of both wild-type and ΔF508 CFTR (Fig. 1) by western blotting, we found that the amount of CFTR protein was greatly reduced in the Cos7 cells transfected with the A455E mutant. To evaluate the degradation of A455E CFTR, we treated the cells with MG132, a non-specific inhibitor of proteasomal degradation. Fig. 2 shows that in the presence of MG132, the protein expression of both the B and C bands of A455E CFTR increased dramatically. A similar effect was seen when we used the more specific proteasome inhibitor, PS341. PS341 also caused an increase in both the B and C bands of A455E. Furthermore, we noted that A455E could be rescued by growing the cells at a reduced temperature, as had previously been observed for ΔF508 [13]. In sharp contrast, there was no increase in the protein expression of A455E CFTR when the cells were treated with the aggresome inhibitor tubacin or the lysosomal inhibitor E64 (Fig. 3). These data suggest that A455E is degraded primarily in proteasomes.

**Figure 4:**
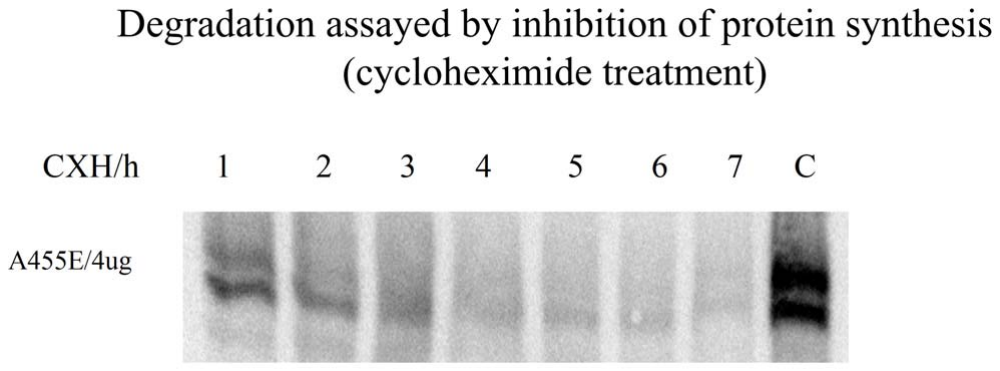
Effect of protein synthesis inhibition on the degradation of A455E. Cos7 cells were transfected with A455E cDNA and treated with cycloheximide (25 µg/ml) for the indicated times. Note the rapid decay of both the mature C and immature B bands of A455E. (n = 4).

To evaluate how rapidly the A455E CFTR protein is degraded, we treated the transfected cells with cycloheximide for between 1 and 7 hours (Fig 4). Surprisingly, both the B and C bands of A455E CFTR rapidly disappeared in the cells treated with cycloheximide (Fig. 4), suggesting that it is rapidly degraded, as is ΔF508 CFTR (see [13]).

### Δ264 CFTR increases the processing of band B to band C of A455E CFTR

**Figure 5:**
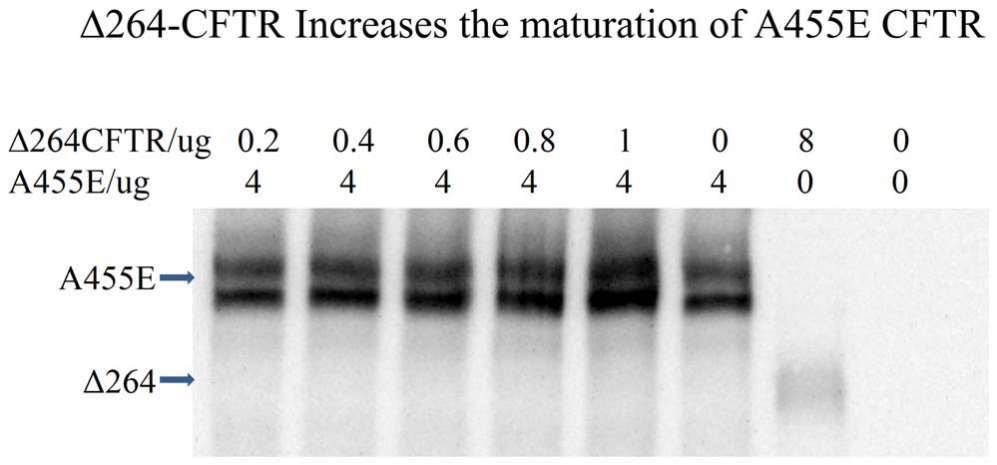
Transcomplementation of A455E by Δ27-264 CFTR . Both the C and B bands of A455E CFTR were increased when cells were cotransfected with Δ27-264 CFTR, showing that A455E could be rescued by transcomplementation. (n = 11).

We then tested whether the truncation mutant, Δ264 CFTR, was capable of transcomplementation with A455E (Fig. 5). In order to determine whether Δ264 CFTR affects the maturation of A455E CFTR, we cotransfected Δ264 CFTR and A455E CFTR into Cos7 cells and found that the mature C band from A455E CFTR was increased in cells cotransfected with Δ264 CFTR, as compared to cells transfected with A455E cDNA alone (Fig. 5).

### Δ264 CFTR binds to A455E CFTR, forming a biomolecular complex

**Figure 6:**
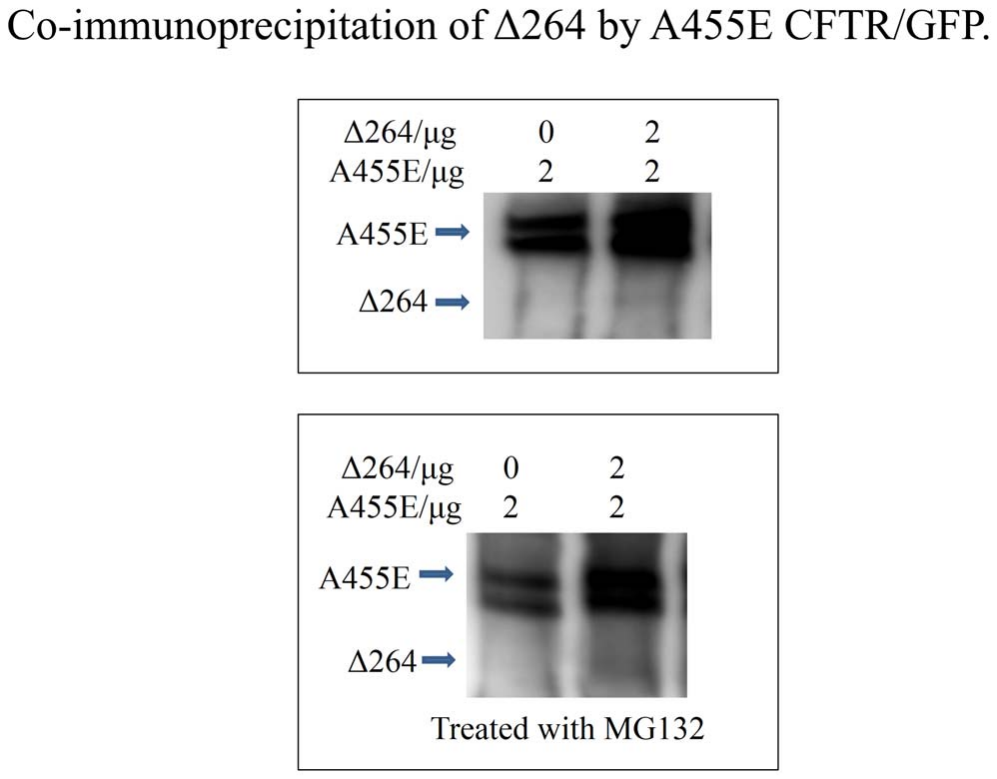
A455E binds to Δ27-264 CFTR. Cos7 cells were transfected with both Δ27-264 CFTR and an A455E CFTR construct bearing a GFP tag. Anti-GFP antibodies were used to pull down A455E, and the gels were blotted with anti-CFTR antibody. (n = 3).

We and others have shown that transcomplementation can occur via direct binding of truncated forms of CFTR to ΔF508-CFTR and via chaperone displacement [17]. In order to assess these possibilities in A455E CFTR, we conducted co-immunprecipitation experiments. Fig. 6 shows that Δ264-CFTR did indeed bind to A455E, in both the absence and presence of the proteasome inhibitor MG123.

### Correctors 4A (C4) and VX325 (C3) increase the processing of band B to band C of A455E CFTR

**Figure 7:**
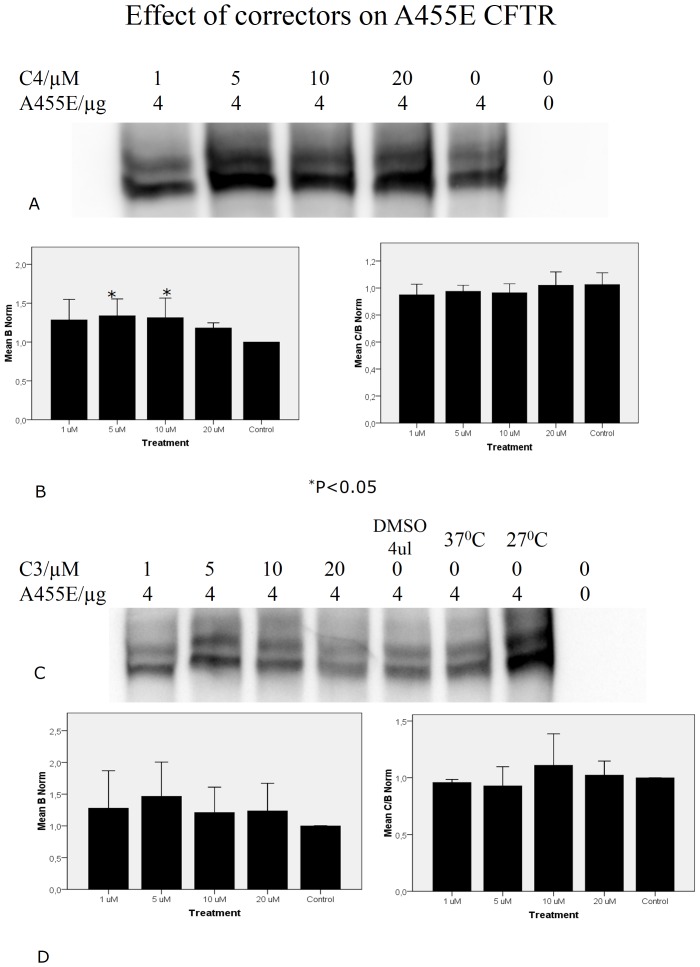
Correctors can rescue A455E. Cos7 cells were transfected with A455E and treated with the correctors C3 or C4 for 16 h at the specified concentrations. Note that C4 had a profound effect on the immature B band of A455E as well as causing an increase in the mature C band (**A, B**). C3 did not have a significant effect (**C, D**). (n = 3).

We next asked whether small-molecule correctors might be effective in rescuing A455E CFTR (Fig. 7). We chose two well-known correctors, 4A (C4) [18] and VX325 (C3) [19]. We found that corrector C4 does have a robust effect on A455E CFTR; in contrast, C3 had only a minimal effect on A455E (Fig. 7).

## Discussion

Transcomplementation of ΔF508-CFTR by fragments of CFTR has been observed [13], [14], [17], [20,20]. These fragments are themselves extremely efficiently degraded; they bind to ΔF508 CFTR and improve the maturation from the immature band B to mature band C. Although transcomplementation has been observed by at least three different groups, it has never been demonstrated with other mutations. Here we show that A455E can also be rescued by transcomplementation. This finding is significant because it provides a new way to treat mutations other than ΔF508 CFTR and also provides insight into the mechanism of transcomplementation.

ΔF508 CFTR is associated with a least two major problems, an unstable NBD1 and defective interactions with intracellular loops (ICL), especially ICL4 [21]–[23]. These two defects disrupt both trafficking and channel activity. The A455E mutation, on the other hand, is located within the F1-type ATP-binding core subdomain near to the ABC protein signature, the Walker A domain [24]. Given the proximity to the Walker A domain, one might expect that A455E would have alterations in gating. However, electrophysiological studies have shown that its single-channel properties are similar to those of wild-type CFTR [11]. A455E also functions well as a regulator of other channels [25]. Thus, its ability to act as a conductance regulator is intact. What appears to be causing the disease, then, is a drastic reduction in the processing of the mature protein. At least two studies have failed to detect mature band C from A455E [11], [26], although the results of their electrophysiological studies suggested that some mature band C must have been present at the plasma membrane in order to generate chloride currents [11]. In our study, we do detect some mature band C at the plasma membrane, but when the cells are treated with cycloheximide to evaluate protein degradation, it is clear that the mature band C of A455E is rapidly degraded along with the immature B band. This situation is similar to that in ΔF508 CFTR, in which both the immature mature bands of temperature-rescued ΔF508 CFTR are rapidly degraded. Van Oene et al. 2000.] have shown that A455E has a pattern of degradation that is clearly different from that of ΔF508 CFTR. A455E appears to be proteolytically cleaved within the NBD1-R domain to form C-terminal aggregates. This cleavage does not appear to occur via the 26S proteasome, but perhaps within the cystosol [26]. Clearly, these results show that the A455E mutant is distinctly different from ΔF508 CFTR.

Nevertheless, A455E shows some similarity to ΔF508 CFTR. Although A455E is uniquely cleaved in the cytoplasm, our results show that it is still degraded in the proteasome, because treatment with two types of proteasome inhibitors led to significant increases in steady-state protein levels. In fact, we saw a rather large increase in the mature band of A455E after proteasome inhibition. This result is in contrast to the response of A455E to lysosomal inhibitors, which were without effect. Inhibiting the aggresome was also without effect. Taken together, our data suggest that A455E, like ΔF508 CFTR, is processed by proteasomes.

Δ264 CFTR rescues A455E and forms a molecular complex between the two molecules. It has been shown that CFTR forms an intramolecular dimer through an interaction between the Walker sites in the two NBDs that involves ATP. Indeed, Lewis et al. [27] have shown that the crystal structure indicates that two isolated NBD1s of CFTR readily form a head-to-tail dimer when both the regulatory insert and extension are removed. It is possible that a similar interaction occurs between Δ264 and A455E. Two other groups have shown that small fragments of CFTR can rescue ΔF508 CFTR, through either a bimolecular interaction [20] or chaperone displacement [17]. One group used a truncation that included TMD1 and NBD1 [20], and the other NBD1 plus the R domain [17]. What these truncations have in common with ours is that each contains NBD1. Thus, it is plausible to suggest that the transcomplementation most likely occurs by an interaction between the NBD1 moieties of the truncated and the mutant proteins. The observation that A455E is readily rescued by Δ264 CFTR suggests that transcomplementation may be influencing NBD1 in the region of the Walker sites. One then may ask why the interaction with the normal NBD2 of A455E does not rescue its own NBD1. One reason may be that NBD2 is translated much later than NBD1 [28]. By the time NBD2 is translated, the mutant NBD1 may already doom the molecule to be degraded by endoplasmic reticulum-associated degradation (ERAD). One might envision that the truncated and mutant CFTRs immediately form a complex during cotranslation of the NBD1s from the two molecules. Thus, the truncated NBD1 could act as a molecular chaperone to rescue the other molecule. Therapeutic transcomplementation to rescue A455E would require gene transfer via a virus or non-viral particle. Several gene therapy approaches are currently being developed for both purposes [29].

Another way to rescue mutant CFTR is with corrector compounds that either bind to mutant CFTR or alter some component of the ERAD pathway. Several groups utilizing high-throughput methods to screen large compound libraries have identified correctors that rescue ΔF508 CFTR. For example, the C4 corrector utilized here was identified in an academic laboratory [30], whereas C3 was developed commercially [19]. Although many compounds have been identified, only VX 809 has reached clinical trials [31]. Although it has shown promise in laboratory experiments in clinical trials involving patients bearing the ΔF508 mutation, VX-809 [31] had a small effect on sweat chloride and no effect on pulmonary function or rescue of ΔF508 CFTR in rectal biopsies. Our demonstration that both C3 and C4 can rescue A455E suggests that A455E CFTR may be a better candidate for correction with either compound correctors or transcomplementation than is ΔF508 CFTR.
